# Baseline assessment of adult and adolescent primary care delivery in Rwanda: an opportunity for quality improvement

**DOI:** 10.1186/1472-6963-13-518

**Published:** 2013-12-17

**Authors:** Ashwin Vasan, Manzi Anatole, Catherine Mezzacappa, Bethany L Hedt-Gauthier, Lisa R Hirschhorn, Fulgence Nkikabahizi, Marc Hagenimana, Aphrodis Ndayisaba, Felix R Cyamatare, Bonaventure Nzeyimana, Peter Drobac, Neil Gupta

**Affiliations:** 1 Partners In Health-Inshuti Mu Buzima, Kigali, Rwanda and Boston, USA; 2 Department of Clinical Research, London School of Hygiene and Tropical Medicine, London, UK; 3 Department of Medicine, Weill Cornell Medical College/New York-Presbyterian Hospital, New York, USA; 4 University of Rwanda, College of Medicine and Health Sciences, School of Public Health, Kigali, Rwanda; 5 Division of Global Health Equity, Brigham and Women’s Hospital, Boston, USA; 6 Department of Global Health and Social Medicine, Harvard Medical School, Boston, USA; 7 Ministry of Health, Government of Rwanda, Kigali, Rwanda

**Keywords:** Primary care, Africa, Resource-limited settings, Quality improvement, Training, Integration, IMAI, Outpatient department, Nurses

## Abstract

**Background:**

As resource-limited health systems evolve to address complex diseases, attention must be returned to basic primary care delivery. Limited data exists detailing the quality of general adult and adolescent primary care delivered at front-line facilities in these regions. Here we describe the baseline quality of care for adults and adolescents in rural Rwanda.

**Methods:**

Patients aged 13 and older presenting to eight rural health center outpatient departments in one district in southeastern Rwanda between February and March 2011 were included. Routine nurse-delivered care was observed by clinical mentors trained in the WHO Integrated Management of Adolescent & Adult Illness (IMAI) protocol using standardized checklists, and compared to decisions made by the clinical mentor as the gold standard.

**Results:**

Four hundred and seventy consultations were observed. Of these, only 1.5% were screened and triaged for emergency conditions. Fewer than 10% of patients were routinely screened for chronic conditions including HIV, tuberculosis, anemia or malnutrition. Nurses correctly diagnosed 50.1% of patient complaints (95% CI: 45.7%-54.5%) and determined the correct treatment 44.9% of the time (95% CI: 40.6%-49.3%). Correct diagnosis and treatment varied significantly across health centers (p = 0.03 and p = 0.04, respectively).

**Conclusion:**

Fundamental gaps exist in adult and adolescent primary care delivery in Rwanda, including triage, screening, diagnosis, and treatment, with significant variability across conditions and facilities. Research and innovation toward improving and standardizing primary care delivery in sub-Saharan Africa is required. IMAI, supported by routine mentorship, is one potentially important approach to establishing the standards necessary for high-quality care.

## Background

The growing success in resource-limited settings of disease-specific interventions for HIV, TB, malaria and other conditions has led to renewed focus on primary care. Integration of disease-specific services into primary care settings has emerged as a strategy to harness specific gains for more generalized improvement in health care delivery, with HIV and reproductive health care most commonly studied [1-7]. Despite this work, relatively little attention has been paid directly to strengthening primary care services for adults and adolescents – the core services with which disease-specific programs should integrate. In many developing-world health systems, particularly in sub-Saharan Africa, the majority of general adult and adolescent ambulatory care occurs in the outpatient department (OPD), which typically operates within front-line health centers (HCs) or as auxiliary clinics to district or regional hospitals. In many settings, the OPD is the first point-of-contact with formalized health infrastructure for patients who are not already enrolled in other specialized ambulatory services (e.g. antenatal or TB care), or care for specific chronic diseases (e.g. HIV). As such, the OPD serves a critical function in the health system through triaging for life-threatening conditions, screening for chronic diseases and modifiable risk factors, diagnosis of acute illness, and appropriate management of both acute and chronic conditions. However, there are currently no benchmarks or targets established to define quality of care for adults and adolescents for most conditions in this primary care setting.

Few rigorous studies have been done on how to develop a standardized, integrated approach across conditions for the initial adult patient screening and work-up in resource-limited settings [8,9]. The few existing studies suggest that standardization and integration could be an important component of improving primary care and health worker performance [10-14], but more research is needed. Aside from disease-specific analyses and one qualitative survey [15] a literature review did not identify any reports describing the quality of general ambulatory primary care for adults and adolescents in any resource-limited setting.

Many tools have been developed for services for *selected* conditions delivered within OPDs, including malaria, STIs, and TB. In addition, an integrated guideline for respiratory disease was developed and tested in southern Africa [16-18]. The World Health Organization (WHO) and partners have attempted to standardize *general* adult and adolescent primary care services, called IMAI – the Integrated Management of Adolescent & Adult Illness [19]. Building off the success of the Integrated Management of Childhood Illness (IMCI) for children under-five years [20], IMAI is targeted for use by front-line health care workers, and consists of simplified, syndromic clinical protocols within a single integrated guideline for common presenting illnesses in adults and adolescents. Like IMCI, these syndromic protocols are organized around symptoms (e.g. fever, cough, diarrhea, etc.) and include a simplified approach to patient history and physical exam. These protocols then provide prescriptive guidance on the management (triage, screening, diagnose, treatment, and follow-up and referral) for a variety of conditions commonly seen in adult outpatient care. Unlike IMCI [21-26], IMAI has undergone little study, with limited, mostly unpublished validations from South Africa Simoes E, et al: *Preliminary Analysis of IMAI Validation Studies.* data.], Ethiopia [27], and Lesotho [Seung KJ, et al: Validation of Integrated Management of Adolescent and Adult Illness (IMAI) guidelines for patients with respiratory symptoms in Lesotho. Unpublished.].

The objective of this study is to describe the routine quality of care for adults and adolescents at OPDs in a rural district in Rwanda in order to document the current quality of care, identify needs for improvement, and propose possible interventions going forward.

## Methods

### Study setting

The study was conducted in southern Kayonza District, in Rwanda’s Eastern Province, with an estimated total population of approximately 190,000 people [28]. The study was conducted at all eight of the Ministry of Health (MOH) health centers that fall under the district hospital catchment area, and all of which receive support from Partners In Health-*Inshuti Mu Buzima* (PIH-IMB). PIH-IMB is a non-governmental organization partnering with the MOH since 2005 to strengthen health systems in three rural districts in Rwanda. Rwandan HCs are generally staffed by nurses holding the equivalent of a secondary school education in nursing. These nurses undergo no specific training in adult and adolescent primary care aside from their degree program and practice experience. There are, however, a number of disease-specific didactic trainings provided by the MOH and partners that some nurses may attend. Routine documentation of delivery of care is done in the OPD register, a standard lined registry that contains basic demographic, diagnostic and treatment data.

### Study population

The study was conducted between February 1, 2011, and March 31, 2011. Consecutive patients 13 years or older presenting to the OPD were eligible for the study. There is no clearly defined age cut-off between childhood and adolescence in Rwanda [29]. Age 13 was chosen for this study based on definitions used by the United States’ Centers for Disease Control (CDC) [30]. Data at each health center were collected over one week, with all centers visited in eight successive weeks.

### Data collection

Data were collected by a clinical mentor, employed by the MOH and assigned to train and supervise OPD nurses as part of a larger initiative to improve quality of care at health centers in the district [31]. Supervision and clinical mentoring activities had not yet begun at the time of baseline data collection. The clinical mentor had the equivalent of a bachelor’s degree in nursing with five years of relevant clinical experience. The clinical mentor underwent extensive didactic and practical training in the adapted IMAI protocols, mentoring and quality improvement. The standard WHO-IMAI protocols [32] were previously adapted and translated by the study staff and district MOH partners through consensus guideline review and pilot testing so as to adhere to Rwandan national treatment guidelines and reflect local epidemiologic priorities.

Data were collected by direct observation of routine clinical consultations using a standard data collection tool developed to document critical components of the clinical encounter. This observation checklist was structured upon the WHO-IMAI Case Management Observation Form, and included the standard triage checklist for emergency conditions contained within the IMAI Quick Check protocol, part of the larger IMAI guideline [19]. IMAI had not been formally introduced in Rwanda at the time of the study and study nurses had not received training in the adapted IMAI protocols.

The clinical mentor was present in the room during the consultation but was instructed to not intervene in patient care, with the exception of a critically ill patient requiring immediate action. Data collected in the observation checklist are summarized in Table 1. Formal diagnostic and management decisions made by the nurse were recorded by the clinical mentor from observed actions and review of the OPD register.

**Table 1:** Summary of data categories within the study Observation Checklist

	
1.	Demographics
2.	Vital Signs
	• Height
	• Weight
	• Pulse
	• Respiratory Rate
	• Temperature
	• Blood pressure
	• BMI (optional)
3.	Triage and Identification of Emergency Conditions
	• Based on IMAI Quick Check triage protocol within WHO-IMAI guideline
4.	Screening
	• Chronic cough
	• Malnutrition
	• Anemia
	• Genital/anal lesions
	• Discharge/other genitourinary symptoms in males with genital lesions
	• Previous HIV testing
	• Mosquito net
	• Tobacco use
	• Alcohol use
	• Sexual activity
	• Pregnancy in eligible females
	• Family planning in females
5.	Chief Complaint(s)
6.	Diagnosis
	• Based on MOH SIS diagnostic codes
7.	Treatment
	• 100% agreement, including labs, medications, and other treatments ordered
8.	Referrals
	• Including follow-up visits to the OPD, if any
	• Patient Age and Sex
	• Start and end time of consultation
	• Health center
	• Nurse education level
	• Years of OPD experience of nurse

The clinical mentor recorded information on up to three chief complaints for each patient consultation. For each patient complaint the nurse, following MOH standards, selected from the 57 MOH *Système d’Information Sanitaire* (SIS) diagnosis codes used in routine practice. The clinical mentor recorded the SIS diagnosis selected by the nurse and selected his own SIS diagnosis based on his clinical expertise and additional training in IMAI.

### Data analysis

The primary outcome of interest was correct diagnosis of patient illness, defined as agreement in diagnosis between the nurse and the clinical mentor, with patient complaint as the unit of observation. For example, a patient complaining of both cough and back pain would receive one diagnosis for each complaint, and contribute two observations to the sample. Cases where the nurse found no appropriate diagnosis within the SIS codes for a given patient complaint were excluded from analysis of diagnosis agreement. Chief complaints were defined as the primary complaint either: (1) offered by the patient voluntarily, (2) given by the patient in response to direct questioning by the nurse, or (3) selected by the nurse independently at any point during the consultation.

Correct treatment of each complaint was also evaluated, defined as 100% agreement between the nurse and clinical mentor in all elements of treatment plan, including labs/diagnostic tests ordered, medications prescribed, referrals or transfers made, and follow-up recommendations. The nurse treatment plan reflected their routine practice, while the clinical mentor’s treatment plan was based on the modified IMAI protocols. Observations where the nurse diagnosis was incorrect were not automatically excluded from the treatment analysis, so as to capture those cases where the nurse may have selected an incorrect diagnosis but still made a correct treatment plan.

Data were double-entered into a Microsoft Access database and analyzed using SASv.9.2 (Cary, NC, USA). The sample was described using frequencies for categorical variables and means for continuous variables. Patient assessment by nurses was described using frequencies of vital sign measurement, triage, and screenings. The primary outcomes – frequencies of correct nurse diagnosis and correct nurse treatment – were calculated for each complaint, along with corresponding 95% confidence intervals. A secondary analysis was conducted to identify factors associated with the quality of nurse case management. Relationships between correct diagnosis and treatment and health center and duration of nurse experience in OPD dichotomized at four years (the sample median) were assessed using Pearson’s chi-squared tests.

### Ethics

This study was approved by the Rwandan National Ethics Committee and the Institutional Review Boards (IRB) at Partners Healthcare and the London School of Hygiene & Tropical Medicine. Data were collected as part of routine program monitoring of an ongoing mentorship and quality improvement intervention in the study district. No identifying nurse or patient information was collected. As such, the IRB approved a waiver of informed consent under the routine use of program monitoring data. However, patients were explained the purpose of the data collection and could opt-out if desired.

## Results

A total of 470 patient consultations were observed (range 53–69 per health center). The median duration of consultation was six minutes (Interquartile Range (IQR): 5–8 minutes) Approximately two-thirds (68.4%) were female and mean patient age was 35.3 years (standard deviation: 15.9 years). All nurses performing the consultations had the nursing education described above. The median practice experience of the nurses was four years (IQR:3–6 years) (Table 2).

**Table 2:** Sample characteristics and distribution of patient complaints

**Number of observed consultations**	**470**
Nurse years of experience in OPD, median (IQR)	4 (3–6)
Duration (minutes), median (IQR)	6 (5–8)
Patient sex, n (%)	
Female	319 (68.4%)
Male	147 (31.6%)
Patient age (years), mean (s.d.)	35.3 (15.9)
Patients asked why they came to OPD, n (%)	61 (13.1%)
Total number of patient complaints	585
Zero (0) complaints	5 (1.1%)
One (1) complaint	354 (75.3%)
Two (2) complaints	102 (21.7%)
Three (3) complaints	9 (1.9%)
Back or joint pain	44 (7.5%)
Cough or difficulty breathing	83 (14.2%)
Diarrhea	31 (5.3%)
Epigastric pain	65 (11.1%)
Fever	58 (9.9%)
Genital or anal sore, ulcer, or wart	5 (0.9%)
Genitourinary symptoms	
Female	88 (15.0%)
Male	32 (5.5%)
Headache or neurological condition	52 (8.9%)
Hypertension	3 (0.5%)
Lower extremity edema	6 (1.0%)
Mental problem	6 (1.0%)
Mouth or throat problem	37 (6.3%)
Skin problem or lump	32 (5.5%)
Other	43 (7.4%)

### Chief complaints

The majority of patients (75.3%) had one chief complaint, with 21.7% (n = 102) reporting two complaints, 1.9% (n = 9) reporting three complaints, and 1.1% (n = 5) with no complaint recorded. The most common chief complaints were female genitourinary issues (15.0%), cough (14.2%), epigastric abdominal pain (11.1%), and fever (9.7%). Of note, only 13.1% (n = 61) of patients were asked directly for a chief complaint by the nurse (Table 2).

### Vital signs

At least one of six vital signs was recorded by any staff at 70.9% of visits, with no visits having all seven vital signs taken (Table 3). At least one vital sign was taken by a registration clerk prior to seeing the nurse in 56.0% of visits, and in 11.5% of patient visits, more than one provider collected vital signs. Weight was the most commonly taken vital sign (62.6%). Temperature was recorded for 27.5% of patients, and 16.2% had blood pressure recorded. No patient had height, respiratory rate or pulse recorded, nor body-mass index (BMI) calculated. One HC had no available registration clerk during any of the patient care sessions observed.

**Table 3:** **Quality Indicators – Consultations with Vital Signs, Triage and Screenings by Outpatient Department nurse (N = 470)**^**a**^

Vital signs collected by:	
Registration clerk	209 (44.5%)
Outpatient Department nurse	118 (25.1%)
Other	75 (16.0%)
Multiple providers	54 (11.5%)
None	14 (3.0%)
Blood pressure recorded	76 (16.2%)
Temperature recorded	129 (27.5%)
Weight recorded	294 (62.6%)
Height recorded	0
Respiratory rate recorded	0
BMI recorded	0
Pulse recorded	0
Triage	
Triaged using basic skills in assessment of severe illness	7 (1.5%)
Diagnosed with emergency condition by nurse	4 (0.9%)
Screening	
Chronic cough	45 (10.0%)
Malnutrition	1 (0.2%)
Anemia	8 (1.8%)
Genital/anal lesions	17 (3.8%)
Discharge/other genitourinary symptoms in males with genital lesions (n = 129)	12 (9.3%) penile
	1 (0.8%) scrotal
Previous HIV testing	7 (1.8%)
Mosquito net	3 (0.7%)
Tobacco use	2 (0.4%)
Alcohol use	2 (0.4%)
Sexual activity	32 (7.3%)
Pregnancy in eligible females (n = 300)	66 (22.0%)
Family planning (n = 268)^b^	10 (3.7%)

^a^unless otherwise indicated.

^b^only women aged 15–49 eligible for screening per adapted IMAI protocol.

### Triage

Only seven (1.5%) patients were fully screened and triaged based on the standard triage protocol within IMAI. Five patients (1%) required higher level of care per the clinical mentor, of whom four were appropriately diagnosed and managed by the nurse (Table 3). All four patient emergencies identified by the nurse were referred to the district hospital; three with high fevers greater than 38°C and one who was convulsing. One patient, identified only by the clinical mentor, was in circulatory shock and with mentor intervention, resuscitation measures were employed.

### Routine screening by history

Low rates were observed for routine screening for a wide range of conditions (Table 3). Only 10% (45) of patients were screened for TB (cough greater than 2 weeks). Among male patients, 10.1% were screened for genitourinary/STI symptoms. Twenty-two percent (66 of 300) of eligible female patients were screened for pregnancy or asked about their last menstrual period. Only 7.3% (32) of patients were asked about current sexual activity, and only 1.8% (7) screened for HIV status. Less than 1% of patients were screened for any of the following: anemia, alcohol use, tobacco use, use of mosquito nets at home or nutrition.

### Diagnosis and treatment

In the 470 consultations observed, patients reported a total of 585 complaints. Of these, forty-nine categorized as “Other” (n = 43) and “Mental problem” (n = 6) were not captured by the disease categories included in the checklist and were excluded from analysis. Seven additional complaints (1.2%) had no corresponding diagnosis in the SIS codes, and 32 (5.5%) were missing any recorded diagnosis information, leaving 497 complaints with a diagnosis available for analysis. In addition to the 49 excluded complaints described above, 33 (5.6%) observations were missing information on correct treatment, leaving 503 complaints with available treatment data for comparison (Table 4).

**Table 4:** Correct nurse diagnosis and treatment of patient illnesses*

		**Nurse diagnosis n = 497**	**Nurse treatment n = 503**
	**N**	**% (95% CI)**^**‡**^	**% (95% CI)**
Total agreement		249	226
		50.1% (45.7 - 54.5)	44.9% (40.6 - 49.3)
Back or joint pain	42	59.5% (44.7 - 74.4)	71.4% (57.8 - 85.1)
Cough or difficulty breathing	80	31.3% (21.1 - 41.4)	33.8% (23.4 - 44.1)
Diarrhea	30	70.0% (53.6 - 86.4)	36.7% (19.4 - 53.9)
Epigastric pain	59	90.0% (82.4 - 97.6)	18.6% (8.7 - 28.6)
Fever	49	8.2% (2.3 - 19.6)	78.6% (67.8 - 89.3)
Genitourinary symptoms			
Female	80	48.8% (38.0 - 59.6)	35.0% (24.6 - 45.5)
Male	30	53.3% (35.5 - 71.1)	26.7% (10.8 - 42.5)
Headache or neurological condition	44	20.5% (8.5 - 32.4)	77.3% (64.9 - 89.7)
Mouth or throat problem	37	70.3% (55.5 - 85.0)	43.2% (27.3 - 59.2)
Skin problem or lump	31	83.9% (70.9 - 96.8)	45.2% (27.6 - 62.7)

*Limited to complaints with n > 5 observations.

^‡^95% CI calculated using the exact binomial distribution.

Nurse diagnoses were in agreement with the clinical mentor 50.1% of the time (95% confidence interval 45.7%–54.5%), with the highest diagnosis agreement seen for epigastric pain (90.0%), skin problems or lumps (83.9%), mouth or throat problems (70.3%), and diarrhea (70.0%). Lowest rates of diagnosis agreement were seen for fever (8.2% correct), headache or neurological conditions (20.5%) and cough or difficulty breathing (31.3%). Agreement varied significantly between HCs, ranging from 35.6% to 60.0% (p = 0.03). There was no association between nurse experience in OPD and correct diagnosis.

The nurse treatment plan was in agreement with the clinical mentor less than half the time (44.9%, 95% confidence interval 40.6% - 49.3%). Complaints of fever (78.6%), headache (77.3%), and back or joint pain (71.4%) had the highest proportion of agreement. Lowest agreement was seen for epigastric pain (18.6%), genitourinary symptoms (26.7% for males, 35.0% for females), cough or difficulty breathing (33.8%), and diarrhea (36.7%). Treatment agreement varied from 32.1% to 58.9% across HCs (p = 0.04), but did not vary with duration of nurse experience in the OPD (Table 5).

**Table 5:** Factors associated with correct nurse diagnosis and treatment of patient illness

		**Nurse diagnosis**		**Nurse treatment**		
	**N**	**Percent correct**	**p**	**N**	**Percent correct**	**p**
Health center			0.03			0.04
A	54	51.9% (38.5 - 65.2)		56	58.9% (46.0 - 71.8)	
B	61	37.7% (25.5 - 49.9)		61	42.6% (30.2 - 55.0)	
C	62	58.1% (45.8 - 70.4)		64	43.8% (31.6 - 55.9)	
D	65	60.0% (48.1 - 71.9)		65	32.3% (20.9 - 43.7)	
E	68	58.8% (47.1 - 70.5)		69	50.7% (38.9 - 62.5)	
F	59	35.6% (23.4 - 47.8)		59	52.5% (39.8 - 65.3)	
G	55	49.1% (35.9 - 62.3)		56	32.1% (19.9 - 44.4)	
H	73	46.6% (35.1 - 58.0)		73	46.6% (35.1 - 58.0)	
Nurse OPD experience			0.26			0.66
4+ years	264	51.5% (45.5 - 57.5)		265	43.8% (37.8 - 49.8)	
0-3 years	220	46.4% (39.8 - 53.0)		225	45.8% (39.3 - 52.3)	

## Discussion

This is, to our knowledge, the first study assessing the general quality of care and clinical decision-making for adults and adolescents at rural OPDs in a resource-limited setting. Every aspect of routine care that was measured, including triage for life-threatening conditions, screening for chronic disease and modifiable lifestyle risk-factors, diagnosis of acute illness, and appropriate management and treatment, were found to be in need of strengthening and quality improvement. The study demonstrated infrequent collection and use of vital signs, infrequent screening for common illnesses (such as HIV, TB, STIs), and low rates of prevention counseling (such as malaria control with ITNs, and alcohol and tobacco use). With a lack of available standards to support quality, compounded by relatively little investment in general primary care for adults and adolescents, it is unsurprising that the results reflect a great need for improvement of front-line delivery.

Nurse diagnosis and treatment decisions were in agreement with the clinical mentor approximately half of the time or less. Agreement in diagnosis and treatment for the same conditions (e.g. fever, epigastric pain) often varied widely, suggesting large variations in individual practice. Poor decision-making could be influenced by a number of long-term structural and health system needs, including gaps in infrastructure, supplies and human resources [33-36]. However, other factors could be addressed in the shorter-term and could lead to an initial rapid improvement in the quality of adult and adolescent primary care.

As noted, aside from selected disease- or condition-specific protocols there are few decision-support tools available to help OPD providers simplify and standardize their decision-making and care. These tools, particularly for children under-5 and for HIV/AIDS, have been shown to improve quality and health worker performance [37-42]. While experiential learning is the primary foundation of practice improvement in Rwandan OPDs, the results show there was no significant increase in the quality of the decision making with increasing years of nursing experience.

The data also highlight needs with respect to nurse training and standardization of care. The low frequencies in our data across the measured areas of clinical practice suggest that the nurses in our sample need additional support in basic clinical skills, including history-taking, completion and interpretation of vital signs, and screening for chronic conditions, in addition to clinical reasoning and decision-making. While it was beyond the scope of this study to isolate whether these gaps originated in pre-service education, pre- or post-service training, and/or other structural factors such as infrastructure, lack of protocols or time constraints, clinical training of nurses in primary care has been shown to at least modestly improve quality of care for acute respiratory tract infections [43] and medication prescription [44]. The data demonstrate that nurses manage some illnesses better than others, and that performance differs significantly between sites, suggesting that standardization of care, as well as performance measurement coupled with mentoring and systems-based quality improvement are needed. In addressing issues of decision support, integrated training, and standardization of care, protocols such as IMAI could represent an important part of a toolkit for developing a common standard of OPD care for adults and adolescents, and improving quality of these services.

While implementation of care based on protocols such as IMAI is necessary, it is not sufficient to support the delivery of high quality of care. A critical component is ensuring durable knowledge and practice improvement through routine and sustainable mentorship of health care workers. Though there are differences in definition and structure of supervision programs [45], mentorship in primary care has been shown to improve and sustain improvements in nurse performance [46-48]. In Rwanda, robust efforts are now underway to implement a system of routine on-site mentorship to health center nurses in all clinical services [31].

There are a number of limitations to our study. First the clinical mentor was not blinded and therefore could have been influenced by observing nurses’ decisions in real-time, thus biasing the results and possibly over-estimating the level of agreement. Having an observer in the room may have also influenced the nurses to perform better or differently than they would in normal circumstances [49]. Given the low level of consistency found, it is unlikely that either potential bias considerably altered the results. Also, clinical decision-making could not be assessed for approximately 15% of patient complaints due to missing data. Approximately half (n = 43) of complaints missing from the analysis were classified as “Other” complaints that did not fall into the conditions selected for study *a priori*. The remainder of missing diagnosis or treatment information (approximately 7% of the total sample) was distributed across complaints and is unlikely to have had meaningful impact on the results. We have also presented clinical decision-making on diagnosis and therapeutics as a proxy for quality of care, but these are only two key components. We did not measure clinical outcomes, nor define current costs, infrastructure, and human resources which all impact or help define quality and which could be pursued in future research. Finally, IMAI has yet to be formally validated, particularly in low-HIV prevalence settings such as Rwanda, which represents an important next step in establishing a standard of general adult and adolescent primary care delivery.

## Conclusions

This study describes the baseline quality of adult and adolescent primary care at Rwandan health center OPDs and demonstrates a clear need for improving the quality and consistency of care. Care based on IMAI or other integrated protocols, accompanied by a robust system of mentorship and supervision that focuses on individual performance as well as systems-level gaps, could be an important approach to simplifying and standardizing the delivery of care for common acute presentations for adults and adolescents in the ambulatory setting. Refining and validating these protocols is needed. Only these coordinated efforts can ensure that high quality and effective primary care is available for all adults and adolescents in resource limited settings.

## Competing interests

All listed authors declare that there are no competing interests – financial or otherwise – with respect to the design or execution of this study, or the writing or publication of the manuscript.

## Authors’ contributions

AV led the principle design of the study including development of data collection tools, data analysis plan, and served as overall co-lead supervisor for the study with MA, and was responsible for writing the first and all subsequent drafts of the manuscript in tandem with MA as well as incorporating authors’ editorial comments. MA was the co-lead supervisor for the study working directly with AV on the study design, and served as the principle field coordinator and field supervisor for the study, refining of data collection tools and techniques, and was responsible with AV for drafting the first and all subsequent drafts of the manuscript, especially the Results and Discussion. CM served as the principal data analyst for the study, developing the study database and leading data entry, and conducted the main data analysis for the study according to the analysis plan, and as well contributing significant inputs to early and all subsequent versions of the manuscript, especially the Methods and Results. BLH was the principle statistician for the study, supporting data collection and analysis, and making significant written and editorial inputs to later versions of the manuscript, especially the Methods, Results, and Discussion. LRH is the Co-Principal Investigator for the larger DDCF research grant, and on this study served both as the senior technical advisor to the lead authors of the study, but also provided significant editorial inputs to all sections of later versions of the manuscript. FN served as the lead district MOH representative on the study and was involved in review and approval of the study protocol and all related study tools and procedures, and played an instrumental role in educating staff at participating sites on the study, while also contributing significant editorial inputs and approval on later drafts of the manuscript. MH was the senior nurse clinical mentor and principal data collector on the study, was involved in development and field-testing of all data tools, and contributed significant editorial inputs to later drafts of the manuscript, especially the Methods section. AN served as the deputy field coordinator for the study under MA and was the principle point of contact for all study staff on the ground and managed all of the study logistics, especially for the data collection and study implementation teams; he also contributed significant editorial inputs and approval for later versions of the manuscript. FRC served as the principle clinical lead on the study, working directly with the nurse mentor during the field testing and data collection stages, and also worked closely with the lead authors on design and field testing of the data collection tools, while also contributing significant editorial inputs and approval for later drafts of the manuscript. BN was the central MOH representative for the study, was instrumental in securing national ethics approval in Rwanda, and was involved particularly at the early stages of the study during design, approval and establishment of district partnership for the study, while also contributing significant editorial inputs to later drafts of the manuscript. PD is also a Co-PI on the larger DDCF grant, and served as the principle organizational lead on the study, involved particularly in the conception and design phases working directly with the lead authors, and provided significant editorial inputs and approval to later drafts of the manuscript, especially the Background, Results, and Discussion. NG served as the senior advisor on the study, involved at all stages of the study working in close collaboration with the lead authors, from design and conception to implementation and data management, and was instrumental in providing editorial inputs and approval to all drafts of the manuscript including the first draft. All authors read and approved the final manuscript.

## Authors’ information

Ashwin Vasan and Manzi Anatole are joint-first authors to this manuscript.

## Pre-publication history

The pre-publication history for this paper can be accessed here:

http://www.biomedcentral.com/1472-6963/13/518/prepub
